# Supplementary material for: Principles of meiotic chromosome assembly revealed in S. cerevisiae
Source: Nat Commun. 2019 Oct 22;10:4795. doi: 10.1038/s41467-019-12629-0 (PMC6805904; doi:10.1038/s41467-019-12629-0)
Supplement: Supplementary file 2 — Supplementary Information [file 41467_2019_12629_MOESM2_ESM.pdf]

Supplementary information for:

Principles of Meiotic Chromosome Assembly Revealed in *Saccharomyces cerevisiae*

Schalbetter, Fudenberg et al. 2019

Supplementary Figures 1-6
Supplementary Tables 1-3

Supplementary Figure 1

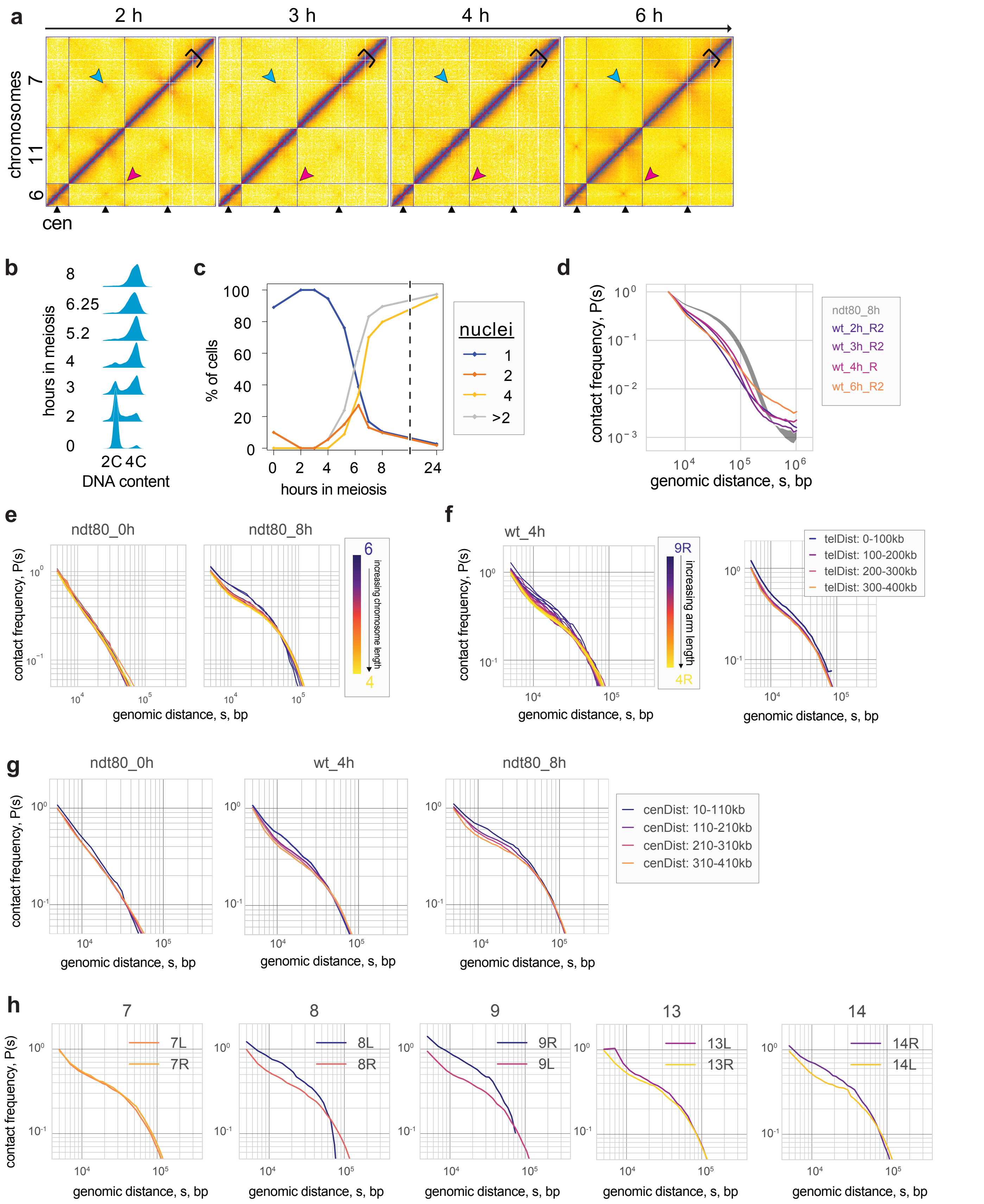

Supplementary Figure 1. Temporal and chromosome length-specific analysis of meiotic chromatin conformation
a-d. Results from a replicate timecourse, collected and characterized independently of the timecourse in Fig. 1. **a.** Hi-C maps, plotted as in Fig. 1a. **b.** FACS to monitor meiotic DNA replication as in Fig. 1b. **c.** DAPI used to monitor meiotic nuclear divisions as in Fig. 1c. Source data are provided as a Source Data file. **d.** *P(s)* used to assess chromosome compaction as in Fig. 1e. **e.** *P(s)* for chromosomes stratified by size for *ndt80Δ* 0 h, *ndt80Δ* 8 h. Short chromosomes display relatively elevated *P(s)* at short distances, and an earlier shoulder. **f.** *Left*: *P(s)* for individual chromosome arms, stratified by size for wild type 4 h. Short arms display relatively elevated *P(s)* at short distances, and an earlier roll-over. *Right*: Intra-arm *P(s)* stratified by the distance from the telomere for wild type 4 h, averaged across all chromosomes. Telomere-proximal regions display elevated *P(s)* at short distances. **g.** Intra-arm *P(s)* stratified by the distance from the centromere for G1 (*ndt80Δ* 0 h), wild type 4 h, *ndt80Δ* 8 h, averaged across all chromosomes. **h.** Contact probability over genomic distance, *P(s)*, of single chromosome arms for *ndt80Δ* 8 h.

Supplementary Figure 2

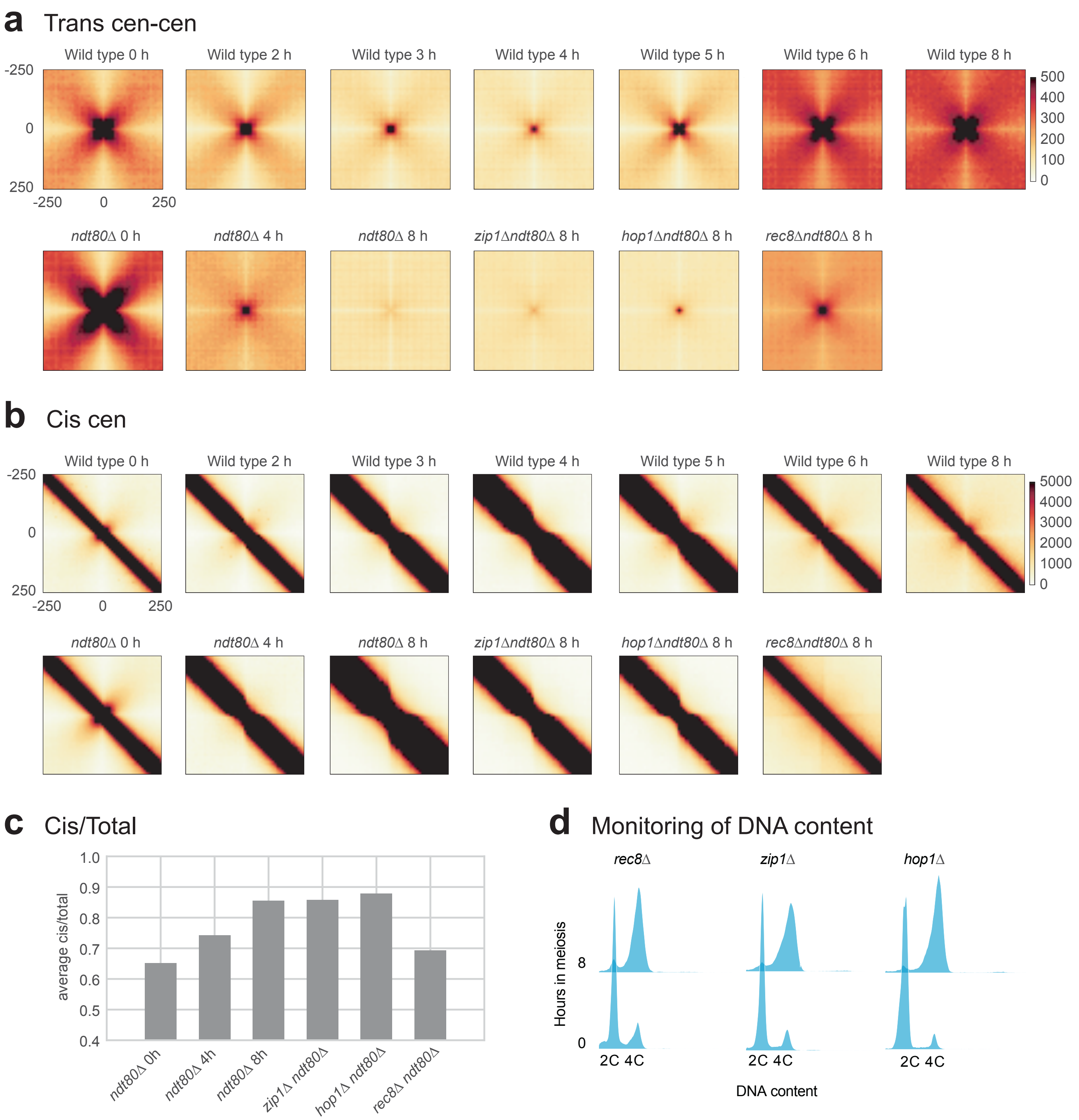

Supplementary Figure 2. Aggregate analysis of centromeric interactions in meiosis
a. Average *trans* centromere-centromere contact maps for indicated data sets. Note that elevated centromere-centromere interactions in *ndt80Δ* as compared to wild type cells are most likely due to technical reasons in cell culture and not a biological effect due to *ndt80Δ*, which is not relevant for vegetative growth[28]. **b.** Average *cis* centromere-centromere contact maps for indicated data sets. Note the loss of the folding back in meiosis, and how the intra-arm enrichment is insulated at centromeres in meiosis. **c.** Average cis/total contact frequency, as in **Fig. 1d**. **d.** FACS analysis for monitoring meiotic DNA replication as in **Fig. 1b**.

Supplementary Figure 3

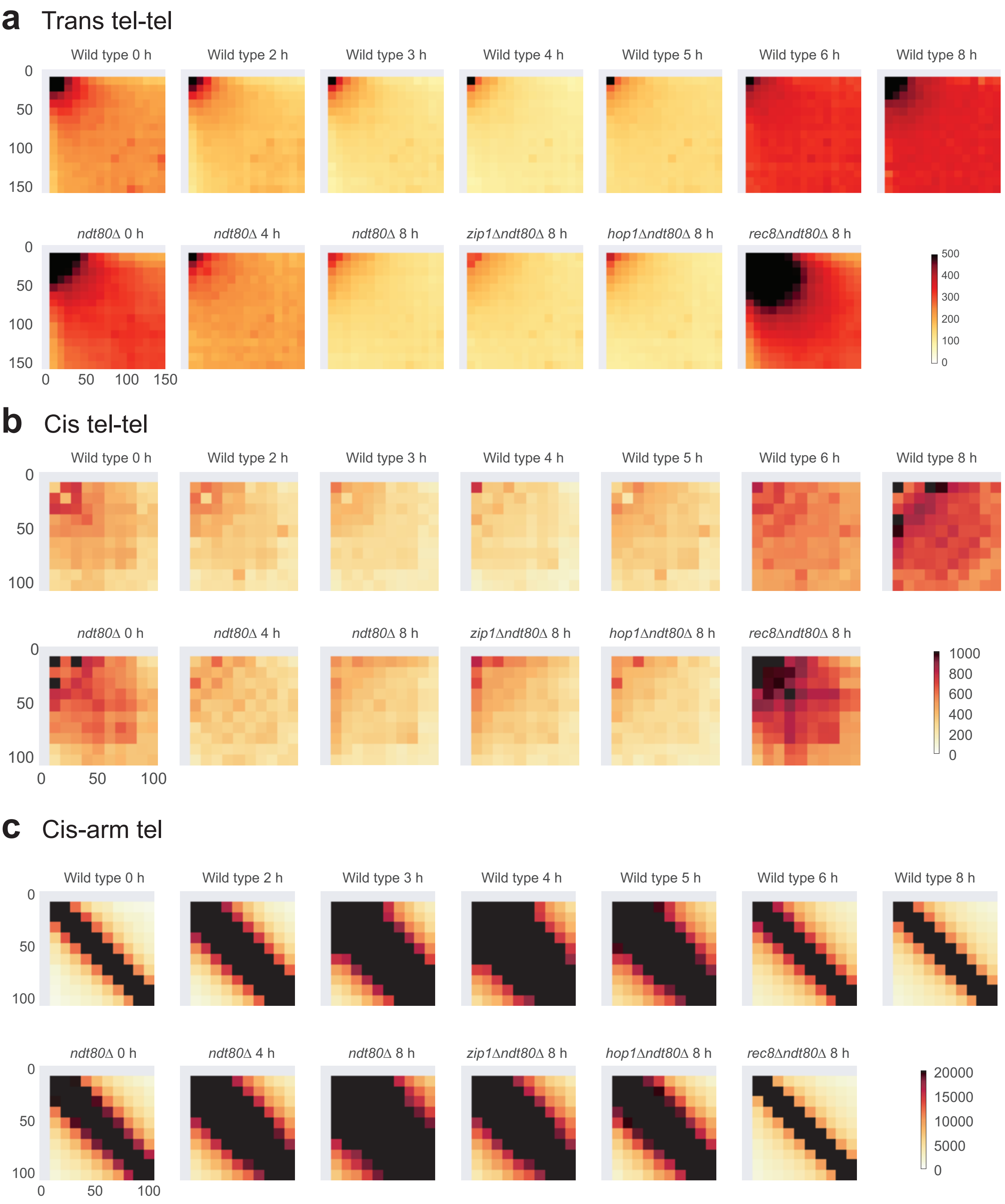

Supplementary Figure 3. Aggregate analysis of telomeric interactions in meiosis

a. Average *trans* telomere-telomere contact maps for indicated datasets.

b. Average telomere-telomere contact maps between the two telomeres of the same chromosome.

c. Average contact map around each telomere in *cis.*

Supplementary Figure 4

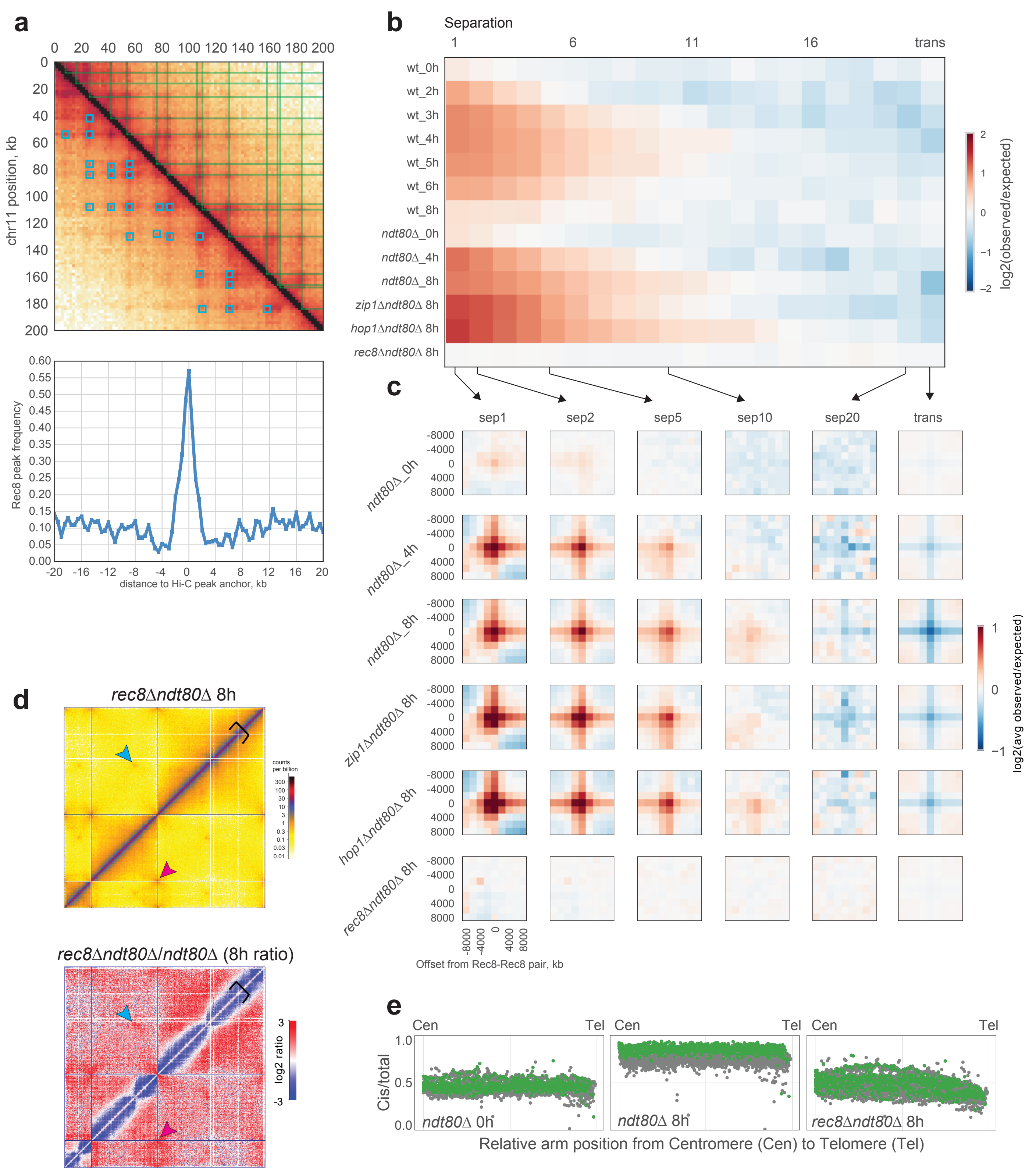

Supplementary Figure 4. Preferred sites of Rec8 occupancy define sites of locus-specific interaction
a. To confirm the correspondence between the pattern of Rec8 binding and Hi-C peaks in meiosis, we performed a reciprocal enrichment analysis. Briefly, we called peaks using the call-dots command line tool in the *cooltools* package (**Methods**), and aggregated Rec8 ChIP peaks around Hi-C peak anchors. As many Hi-C peaks have shared starting or end points, this calculation used a set of unique anchors to avoid double counting. *Top:* 200kb region of chr11 *ndt80Δ* 8h, overlaid with positions of called Hi-Cpeaks (blue dots) and rec8 sites (green lines). *Bottom:* Frequency of Rec8 sites around Hi-C anchors, using 500bp bin size. **b.** Log2 observed over expected contact frequency at Rec8-Rec8 peak pairs as a function of separation across datasets. **c.** Log2 observed over expected contact frequency ±8 kb around Rec8-Rec8 peak pairs at the indicated separations. Together, **b-c** demonstrate that Rec8-Rec8 enrichments are strongest between adjacent sites, decrease between non-adjacent sites with increasing genomic separation, and are absent in *trans*. Equally important, these meiotic features are lost in *rec8Δ*. As for mammalian interphase, this observation in meiosis argues for a *cis*-acting process underlying the formation of focal interactions between Rec8 sites. **d.** *Left*: Hi-C contact maps of *rec8Δ ndt80Δ*. Chromosomes 6, 11 and 7 are shown as representatives for the whole genome. *Right*: Log2 Hi-C ratio maps of *rec8Δ ndt80Δ* / *ndt80Δ*. Plotted as in Fig. 1g. **e.** cis/total as a function of distance along the chromosomal arm, Rec8 sites marked in green.

Supplementary Figure 5

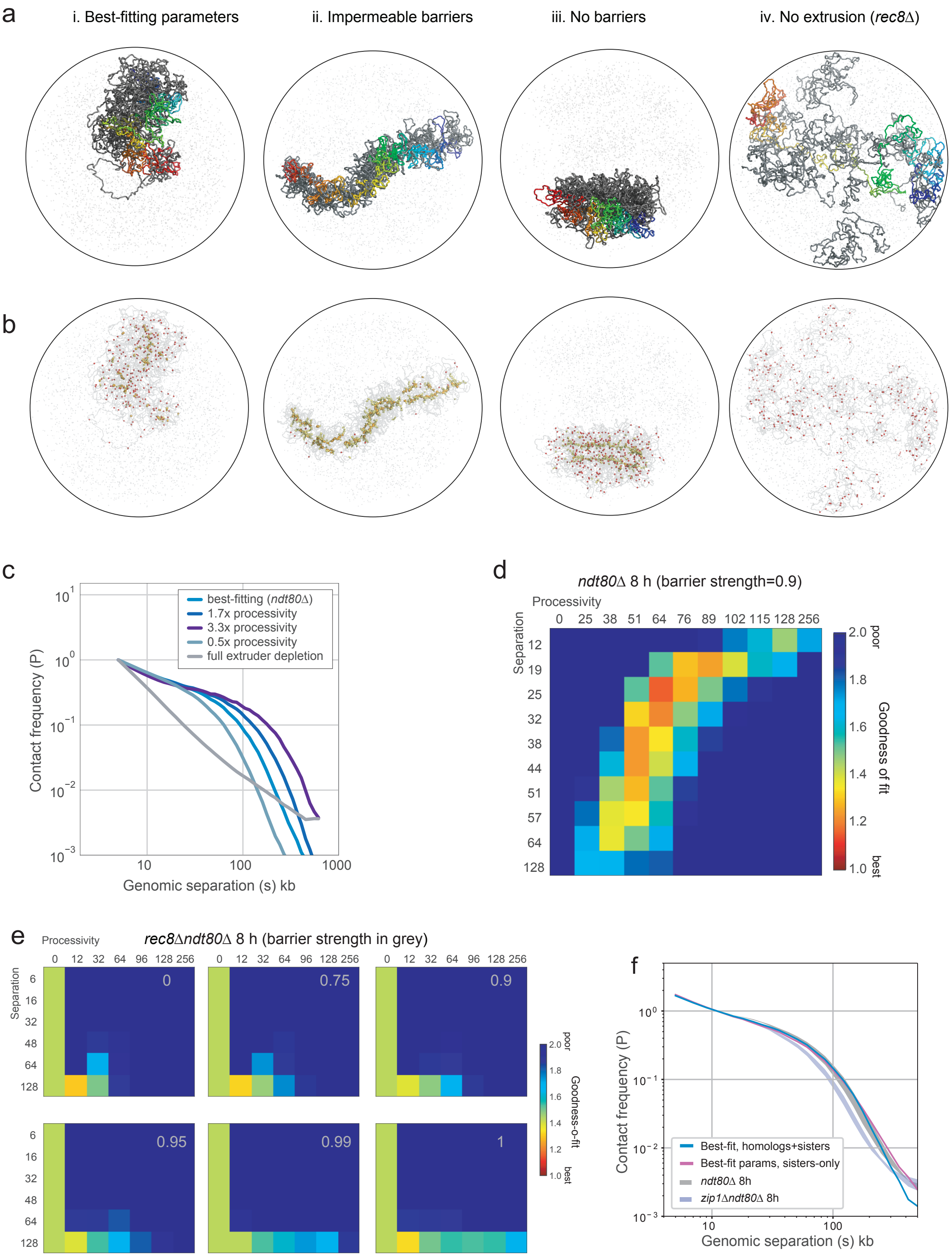

Supplementary Figure 5. Polymer simulations of loop extrusion reveal best-fitting parameters and conformations
a. Representative conformation for the indicated parameter sets. As in Fig. 4a, one chromatid from a homologous quartet of chromatids colored from start to end according to the spectrum; other three colored in grey. **b.** For the same four conformations, positions of Rec8 sites indicated with red spheres, positions of extruded loop bases in yellow, and extruders overlapping a Rec8 site in orange. Note the stable loops between neighboring Rec8 sites creates a very elongated chromatid (ii). Also note the majority of Rec8 sites are unoccupied in (iii), despite the self-assembly of two axial cores and a strong brush. Finally, note very dispersed chromosomes in (iv), consistent with EM[3] for *rec8*Δ. **c.** Contact frequency versus distance, *P(s),* for indicated simulations. Note that the loss of the shoulder in *P(s)* in the case of full extruder depletion mirrors the difference between experimental *ndt80*Δ and *rec8*Δ Hi-C maps. Simulations with increased processivity predict that *P(s)* would shift rightward if unloading was impaired, as could happen in *wapl*Δ. Conversely, if unloading was enhanced, simulations with decreased processivity indicate a leftward shift in P(s), until the absence of extruders. **d.** Goodness-of-fit for a fine grid of processivity versus separation at barrier strength 0.90. The best-fit occurs at similar processivity and separation as for barrier strength 0.95 shown in Fig. 3c, but with slightly lower goodness-of-fit. **e.** Goodness-of-fit to *rec8*Δ data for simulations with the indicated barrier strengths (in grey: 0.00, 0.75, 0.90, 0.95, 0.99, 1.00) over coarse grids of processivity and separation demonstrates that the best fits have few if any extruded loops, regardless of barrier strength. Note that parameters in best agreement with experimental *ndt80*Δ Hi-C data fit the experimental *rec8*Δ Hi-C data poorly (Average log(fold deviation)>2). **f.** *P(s)* curves for simulations with sisters and homologues with the best-fitting parameters for *ndt80*Δ*-8h* maps compared to P(s) for simulations with sisters only show that simply removing homologue tethering does not recapitulate the sort of shifted P(s) seen experimentally in *zip1*Δ Hi-C.

Supplementary Figure 6

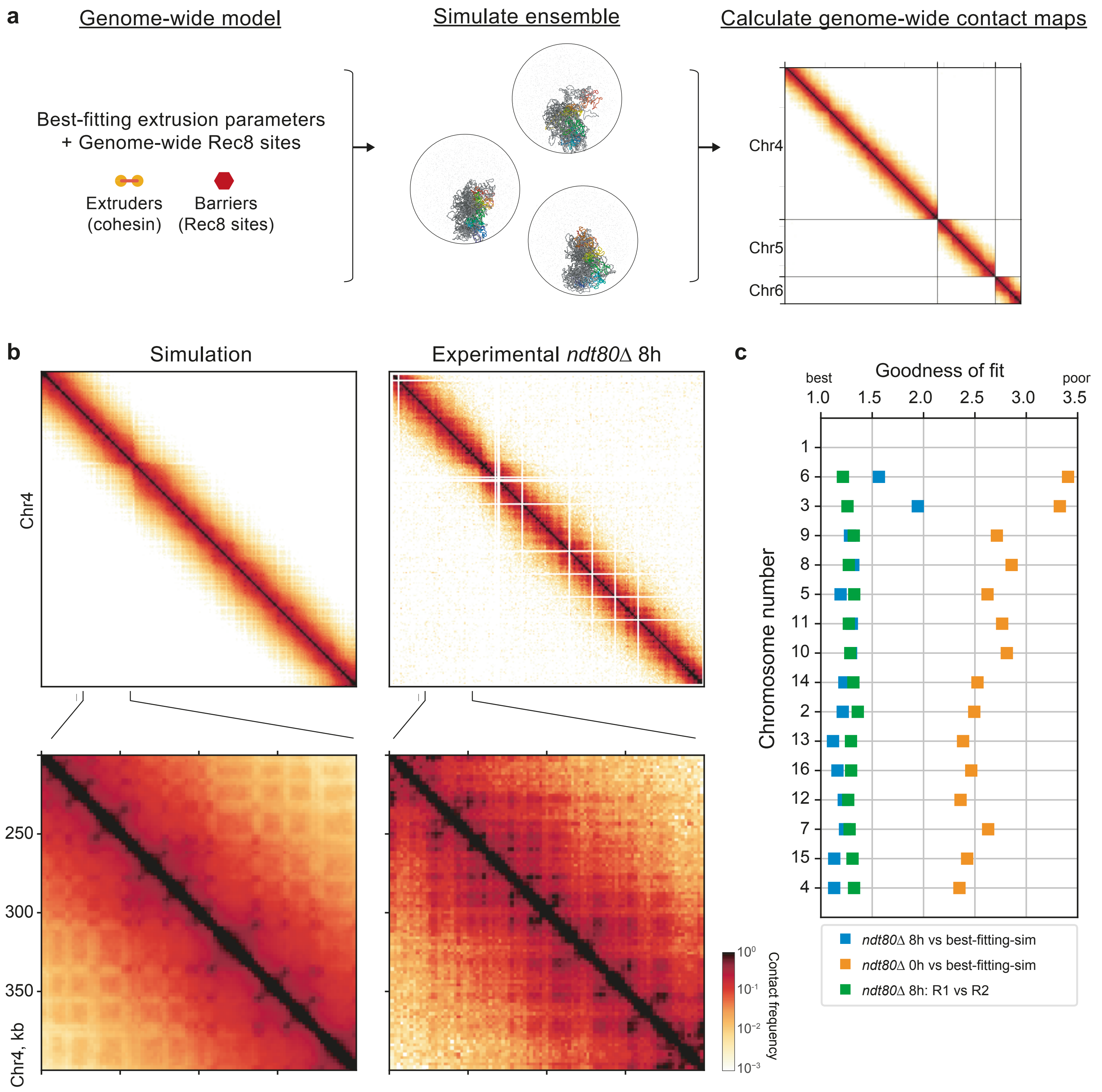

Supplementary Figure 6. Genome-wide simulations of loop extrusion with the best-fitting parameters.
a. To test the transferability of our model across chromosomes, we ran simulations for best-fitting loop extrusion parameters found for chromosome 13 for the full genome, representing each chromosome as a polymer fibre and taking positions of barriers for Rec8 sites across the genome (*left*). Using these parameters we simulated an ensemble of conformations (*middle*) and calculated genome-wide heatmaps at 2 kb resolution (*right*). **b.** Contact maps for chromosome 4 and for a zoomed 200 kb region of chromosome 4 for simulations (*left*) and experimental data (*right*). **c.** Goodness-of-fit on a per-chromosome basis, with chromosomes ordered by length, calculated as previously in **Fig. 3** for chromosome 13. Blue dots show the goodness-of-fit for simulations versus combined experimental *ndt80Δ* 8 h replicates, green dots show the goodness-of-fit for replicates, and orange dots show goodness-of-fit between meiotic simulations and G1 data. Note that as for other analyses, chromosome 1 was excluded as few informative bins remained after filtering. Also note that the shortest chromosomes, chromosome 3 and chromosome 6, fit relatively poorly. Together this analysis shows that for most chromosomes, the goodness-of-fit between simulated and experimental data (blue) shows similar quantitative agreement as found previously for chromosome 13 (~1.11). These values approach the level of agreement between biological replicates (green), and are substantially lower than the discrepancy with G1 data (orange).

Supplementary Tables

Supplementary Table 1. *S. cerevisiae* strains used in this study

Strain name	genotype
MJ6	*ho::LYS2/", lys2/", ura3/", arg4-nsp/", leu2::hisG/", his4X::LEU2/", nuc1::LEU2/"*
SSY14	*ho::LYS2/", lys2/", ura3/", arg4-nsp/", leu2::hisG/", his4X::LEU2/", nuc1::LEU2/", ndt80Δ::LEU2/"*
SSY20	*ho::LYS2/", lys2/", ura3/", arg4-nsp/", leu2::hisG/", rec8Δ::KanMX4/", ndt80Δ::LEU2/"*
SSY25	*ho::LYS2/", lys2/", ura3/', arg4-nsp/", leu2::hisG/", his4X::LEU2/", nuc1::LEU2/", zip1::LEU2/", ndt80Δ::LEU2/"*
SSY49	*ho::LYS2/", lys2/", ura3/", arg4-nsp/", leu2::hisG/", nuc1::LEU2/", his4X::LEU2/", hop1::LEU2/", ndt80Δ::LEU2/"*
SSY58	*ho::hisG/", lys2/", ura3/", leu2::hisG/", nuc1::LEU2/", arg4-nsp/", rec8::KanMX/", ndt80Δ::LEU2/"*

Supplementary Table 2. Overview of proteins described in this study

**Protein**	**Description**
Ndt80	Transcription factor required for exit from pachytene
Rec8	Meiosis-specific kleisin subunit of cohesin
Hop1	Axial element of the synaptonemal complex
Zip1	Transverse filament of the synaptonemal complex

Supplementary Table 3. Hi-C Libraries

**Name**	**Mutations**	**Sample name**	**Valid pairs (M)**
**Main figures**			
wt-0h/G1		HiC_MJ6_wt_2A_0h	14.5
wt-2h		HiC_MJ6_wt_2A1_2h	27.6
wt-3h		HiC_MJ6_wt_2A_3h	24.1
wt-4h		HiC_MJ6_wt_2A_4h	28
wt-5h		HiC_MJ6_wt_2A1_5h	27.6
wt-6h		HiC_MJ6_wt_2A1_6h	27.6
wt-8h		HiC_MJ6_wt_2A3_8h	19
rec8Δ	*rec8Δ ndt80Δ*	average	
rec8Δ replica 1	*rec8Δ ndt80Δ*	HiC_SSY20_ndt80Drec8D_1A2_8h	39.3
rec8Δ replica 2	*rec8Δ ndt80Δ*	HiC_SSY58_ndt80Drec8D_2A_8h	20.2
ndt80Δ	*ndt80Δ*	average, 8h	
G1	*ndt80Δ*	HiC_SSY14_ndt80D_1A2_0h	36
ndt80Δ-4h	*ndt80Δ*	HiC_SSY14_ndt80D_1A_4h	11.9
ndt80Δ replica 1	*ndt80Δ*	HiC_SSY14_ndt80D_1A1_8h	22.9
ndt80Δ replica 2	*ndt80Δ*	HiC_SSY14_ndt80D_2A2_8h	37
zip1Δ	*zip1Δ ndt80Δ*	average	
zip1Δ replica 1	*zip1Δ ndt80Δ*	HiC_SSY25_ndt80Dzip1D_1B2_8h	22.7
zip1Δ replica 2	*zip1Δ ndt80Δ*	HiC_SSY25_ndt80Dzip1D_2A_8h	28.6
hop1Δ	*hop1Δ ndt80Δ*	hop1 ndt80	
hop1Δ replica1	*hop1Δ ndt80Δ*	HiC_SSY49_ndt80Dhop1D_1A_8h	32.8
wt mitotic replica 1		HiC_MJ6_wt_4A1_noco	51.6
wt mitotic replica 2		HiC_MJ6_wt_5A1_noco	82.3
**Supplementary Figures**			
wt-2h		HiC_MJ6_wt_3A_2h	22.5
wt-3h		HiC_MJ6_wt_3A_3h	19.8
wt-4h		HiC_MJ6_wt_3A_4h	16.7
wt-6h		HiC_MJ6_wt_3A_6h	37.6
