# Supplementary material for: Principles of meiotic chromosome assembly revealed in S. cerevisiae
Source: Nat Commun. 2019 Oct 22;10:4795. doi: 10.1038/s41467-019-12629-0 (PMC6805904; doi:10.1038/s41467-019-12629-0)
Supplement: Supplementary file 3 — Reporting Summary [file 41467_2019_12629_MOESM3_ESM.pdf]

nature research

Corresponding author(s): Matthew J Neale

Last updated by author(s): Sep 12, 2019

# Reporting Summary

Nature Research wishes to improve the reproducibility of the work that we publish. This form provides structure for consistency and transparency in reporting. For further information on Nature Research policies, see Authors & Referees and the Editorial Policy Checklist.

## Statistics

For all statistical analyses, confirm that the following items are present in the figure legend, table legend, main text, or Methods section.

| n/a | Confirmed | |
|---|---|---|
| ☐ | ☒ | The exact sample size (*n*) for each experimental group/condition, given as a discrete number and unit of measurement |
| ☐ | ☒ | A statement on whether measurements were taken from distinct samples or whether the same sample was measured repeatedly |
| ☒ | ☐ | The statistical test(s) used AND whether they are one- or two-sided<br>*Only common tests should be described solely by name; describe more complex techniques in the Methods section.* |
| ☒ | ☐ | A description of all covariates tested |
| ☒ | ☐ | A description of any assumptions or corrections, such as tests of normality and adjustment for multiple comparisons |
| ☒ | ☐ | A full description of the statistical parameters including central tendency (e.g. means) or other basic estimates (e.g. regression coefficient) AND variation (e.g. standard deviation) or associated estimates of uncertainty (e.g. confidence intervals) |
| ☒ | ☐ | For null hypothesis testing, the test statistic (e.g. *F*, *t*, *r*) with confidence intervals, effect sizes, degrees of freedom and *P* value noted<br>*Give P values as exact values whenever suitable.* |
| ☒ | ☐ | For Bayesian analysis, information on the choice of priors and Markov chain Monte Carlo settings |
| ☒ | ☐ | For hierarchical and complex designs, identification of the appropriate level for tests and full reporting of outcomes |
| ☒ | ☐ | Estimates of effect sizes (e.g. Cohen's *d*, Pearson's *r*), indicating how they were calculated |

*Our web collection on statistics for biologists contains articles on many of the points above.*

## Software and code

Policy information about availability of computer code

| | |
|---|---|
| Data collection | N/A |
| Data analysis | Open source software available here: https://bitbucket.org/mirnylab/openmm-polymer<br>https://bitbucket.org/mirnylab/mirnylib, https://github.com/mirnylab/cooltools |

For manuscripts utilizing custom algorithms or software that are central to the research but not yet described in published literature, software must be made available to editors/reviewers. We strongly encourage code deposition in a community repository (e.g. GitHub). See the Nature Research guidelines for submitting code & software for further information.

## Data

Policy information about availability of data

All manuscripts must include a data availability statement. This statement should provide the following information, where applicable:

- Accession codes, unique identifiers, or web links for publicly available datasets
- A list of figures that have associated raw data
- A description of any restrictions on data availability

Raw sequence reads are accessible via the SRA repository GSE127940. Code used to analyse Hi-C data publicly available online: https://bitbucket.org/mirnylab/mirnylib, https://github.com/mirnylab/cooltools. Code used to develop polymer simulations publicly available online: https://bitbucket.org/mirnylab/openmm-polymer/. Hi-C matrices publicly viewable via the interactive HiGlass viewer{Kerpedjiev et al., 2018, #17865}, hosted at http://higlass.pollard.gladstone.org. Data monitoring the cell culture—evaluation of meiotic progression by DAPI (Fig. 1c; Supplementary Data Fig. 1c) and FACS (Fig. 1b,f; Supplementary Data Fig. 1b; Supplementary Data Fig. 2d)—are available in the Source Data File (Source_Data.xls) and as a .zip folder, respectively.

# Field-specific reporting

Please select the one below that is the best fit for your research. If you are not sure, read the appropriate sections before making your selection.

☒ Life sciences ☐ Behavioural & social sciences ☐ Ecological, evolutionary & environmental sciences

For a reference copy of the document with all sections, see nature.com/documents/nr-reporting-summary-flat.pdf

# Life sciences study design

All studies must disclose on these points even when the disclosure is negative.

| | |
|---|---|
| Sample size | Biological replicates were performed |
| Data exclusions | Not applicable |
| Replication | All data were suitably replicable |
| Randomization | Not applicable |
| Blinding | Not applicable |

# Reporting for specific materials, systems and methods

We require information from authors about some types of materials, experimental systems and methods used in many studies. Here, indicate whether each material, system or method listed is relevant to your study. If you are not sure if a list item applies to your research, read the appropriate section before selecting a response.

## Materials & experimental systems

| n/a | Involved in the study | |
|---|---|---|
| ☒ | ☐ | Antibodies |
| ☒ | ☐ | Eukaryotic cell lines |
| ☒ | ☐ | Palaeontology |
| ☒ | ☐ | Animals and other organisms |
| ☒ | ☐ | Human research participants |
| ☒ | ☐ | Clinical data |

## Methods

| n/a | Involved in the study | |
|---|---|---|
| ☒ | ☐ | ChIP-seq |
| ☐ | ☒ | Flow cytometry |
| ☒ | ☐ | MRI-based neuroimaging |

## Flow Cytometry

### Plots

Confirm that:

☒ The axis labels state the marker and fluorochrome used (e.g. CD4-FITC).

☒ The axis scales are clearly visible. Include numbers along axes only for bottom left plot of group (a 'group' is an analysis of identical markers).

☒ All plots are contour plots with outliers or pseudocolor plots.

☒ A numerical value for number of cells or percentage (with statistics) is provided.

### Methodology

| | |
|---|---|
| Sample preparation | Cells were fixed in 70% EtOH, digested with 1 mg/ml RNAse (10 mM Tris-HCl pH 8.0, 15 mM NaCl, 10 mM EDTA pH 8.0) for 2 h at 37 ˚C, 800 rpm and subsequently treated with 1 mg/ml Proteinase K in 50 mM Tris-HCl pH 8.0 at 50 ˚C, 800 rpm for 30 min for analysis by FACS. Cells were then washed in 50 mM Tris-HCl pH 8.0 and stained in the same buffer with 1 µM Sytox green or 1 µg/ml Propidium Iodide (PI) overnight in the fridge. |
| Instrument | Accuri C6 |
| Software | Acquirement Accuri C6 software<br>FACS profiles were plotted with R using the library hwglabr2 (https://github.com/hochwagenlab/hwglabr2), applying the following gates: For Sytox green (gate=c(200000,3000000)) and for PI (gate=c(800,10000)) |
| Cell population abundance | NA |

Gating strategy

FACS profiles were plotted with R using the library hwglabr2 (https://github.com/hochwagenlab/hwglabr2), applying the following gates: For Sytox green (gate=c(200000,3000000)) and for PI (gate=c(800,10000))

☒ Tick this box to confirm that a figure exemplifying the gating strategy is provided in the Supplementary Information.
